# Pyrophosphate metabolism and calcification

**DOI:** 10.18632/aging.101703

**Published:** 2018-12-07

**Authors:** Magda R. Hamczyk, Ricardo Villa-Bellosta

**Affiliations:** 1 Departamento de Bioquímica y Biología Molecular, Universidad de Oviedo, Oviedo, Spain; 2 Instituto de Investigación Sanitaria de la Fundación Jiménez Díaz, Madrid, Spain

**Keywords:** phosphate, pyrophosphate, vascular calcification, TNAP

Vascular calcification is associated with physiological aging; genetic diseases, such as Hutchinson-Gilford progeria syndrome and generalized arterial calcification of infancy; and various pathological conditions, such as chronic kidney disease and diabetes [1]. Vascular calcification is characterized by the deposition of calcium-phosphate crystals in the aortic media (called “Monckeberg’s medial sclerosis”) and/or intima (related to atherosclerosis), usually as hydroxyapatite, the main component of bone. Vascular calcification reduces aortic and arterial compliance and elastance, hampering cardiovascular system function. It is linked to poor clinical outcomes and contributes to cardiovascular morbidity and mortality. Because tissue mineralization may occur at normal concentrations of calcium and phosphate, regulatory mechanisms exist to limit this process to bone and cartilage. Several endogenous inhibitors of vascular calcification have been identified, including the matrix Gla protein, fetuin A, osteopontin, and pyrophosphate [1].

Pyrophosphate is a potent inhibitor of calcium-phosphate crystal formation and growth [2]. Extracellular pyrophosphate is produced upon ATP hydrolysis by the enzyme ectonucleotide pyrophosphatase/phosphodiesterase 1 (eNPP1) [3]. Pyrophosphate is degraded to phosphate by tissue-nonspecific alkaline phosphatase (TNAP), promoting calcification [4]. Another enzyme, ectonucleoside triphosphate diphosphohydrolase 1 (eNTPD1), can hydrolyze ATP and ADP to phosphate, reducing the availability of ATP for pyrophosphate production and likely inducing calcification.

Vascular tissue mineralization occurs when the synthesis of vascular calcification inhibitors is impaired or when the formation of calcium-phosphate crystals is enhanced, for example, by hyperphosphatemia, the main risk factor for vascular calcification [2]. Hyperphosphatemia also induces osteochondrogenic phenotypic transition in vascular smooth muscle cells, resulting in the increased expression of bone morphogenetic protein 2 (BMP2) and the reduced expression of adult smooth muscle marker 22α (SM22α) [5].

Despite findings showing that hyperphosphatemia triggers vascular calcification, the effects of hyperphosphatemia on extracellular pyrophosphate metabolism remain unclear. A recent study [6] investigated pyrophosphate metabolism in the context of phosphate-induced vascular calcification. First, this study compared various methods of detecting calcium-phosphate deposition, finding that use of 45-calcium as a radiotracer could identify micro-calcifications, whereas von Kossa and Alizarin Red stains, widely used in studies of calcification, were only capable of detecting advanced stages of calcification. Second, an *ex vivo* model of phosphate-induced calcification showed that devitalized aortas calcify faster than aortas containing living cells. These results confirmed the findings of a previous *in vitro* study with live and dead vascular smooth muscle cells, which showed that calcification is a passive process that can be actively prevented by pyrophosphate [3,7].

**Figure 1:**
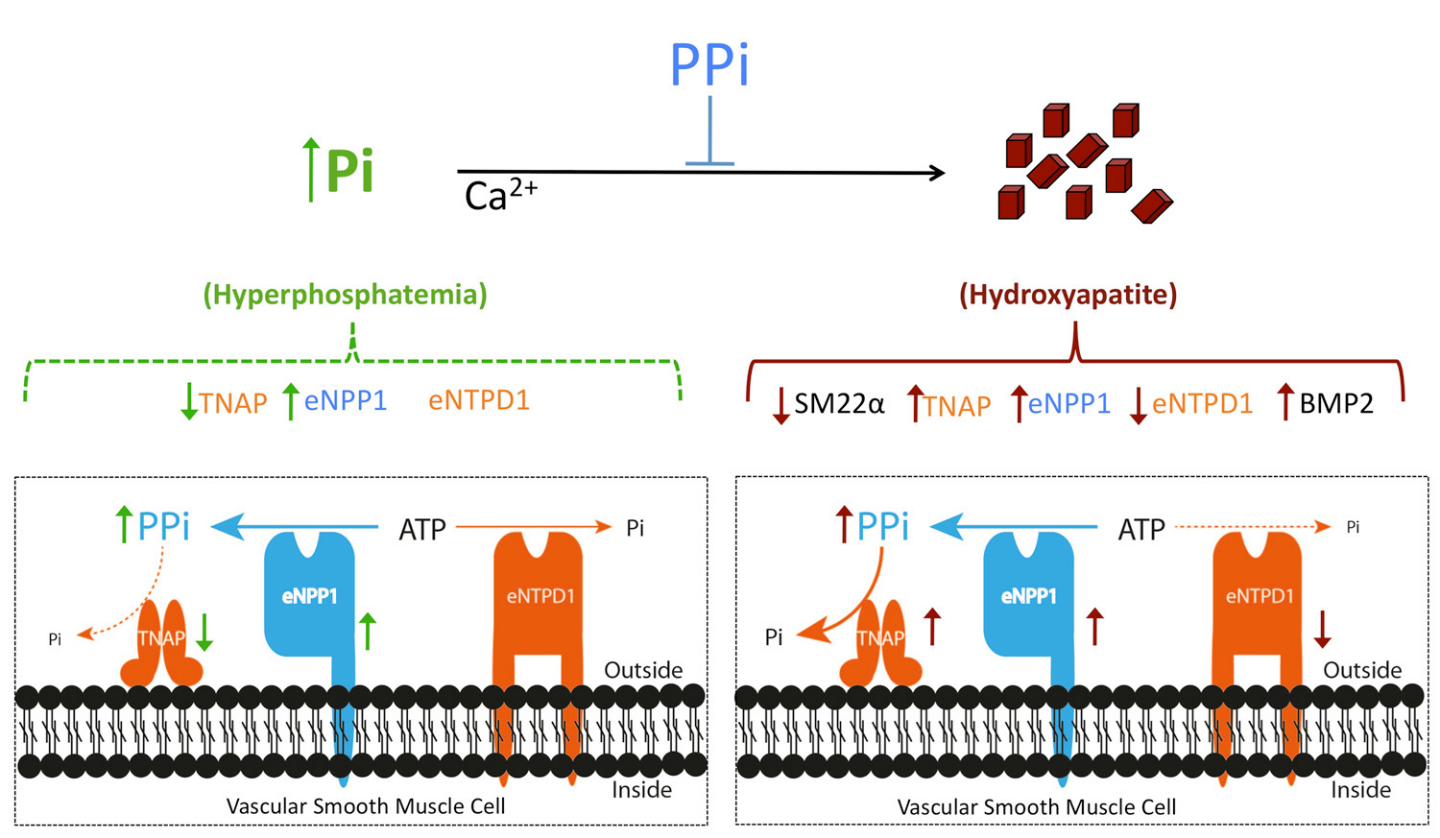
**Extracellular pyrophosphate metabolism during phosphate-induced calcification.** Extracellular pyrophosphate (PPi) is produced upon ATP hydrolysis by the enzyme ectonucleotide pyrophosphatase/phosphodiesterase 1 (eNPP1). Pyrophosphate is degraded to phosphate (Pi) by tissue-nonspecific alkaline phosphatase (TNAP). Ectonucleoside triphosphate diphosphohydrolase 1 (eNTPD1), hydrolyzes ATP (and ADP) to Pi. Figure shows changes in the extracellular pyrophosphate metabolism in response to high concentrations of Pi (before hydroxyapatite is formed) and in response to hydroxyapatite. Hydroxyapatite also increases the expression of bone morphogenetic protein 2 (BMP2) and reduces the expression of adult smooth muscle marker 22α (SM22α).

Finally, we showed that there are two responses to high phosphate concentration (Figure 1): early (before calcium-phosphate crystals are formed) and late (when crystals are already present). During the early response, the pro-calcifying enzyme TNAP is inhibited and the anti-calcifying enzyme eNPP1 is upregulated, providing a compensatory mechanism that can prevent calcium-phosphate crystal deposition. If the early response is faulty, however, in that crystal formation cannot be inhibited and hydroxyapatite accumulates, the late response begins, in which TNAP and eNPP1 are upregulated and the pro-calcifying enzyme eNTPD1 is downregulated. Accordingly, vascular smooth muscle cells incubated with hydroxyapatite show increased expression of both TNAP and eNPP1 and decreased expression of eNTPD1. Interestingly, hydroxyapatite also increases the expression of BMP2 [6–8] and reduces the expression of SM22α [6,7].

These results may explain the contradictory results showing that TNAP expression is increased or decreased during vascular smooth muscle cell calcification [4,8]. Nevertheless, the contribution of TNAP (pyrophosphate→phosphate) to vascular calcification may be limited because the rate of pyrophosphate hydrolysis is approximately 10 times slower than the rate of pyrophosphate synthesis [6]. Accordingly, inhibition of TNAP activity during ATP hydrolysis did not significantly alter pyrophosphate production in the experimental models used in this study. Thus, pyrophosphate production is mainly determined by the ratio of eNPP1 (ATP→pyrophosphate) to eNTPD1 (ATP→phosphate) expression and activity [6]. The main conclusion of this new study was that high phosphate concentrations resulted in the increased synthesis of pyrophosphate over time by both vascular smooth muscle cells *in vitro* and rat aortas *ex vivo*. Moreover, the hydrolysis of pyrophosphate was found to decrease during early stages, but increase during later stages, of hyperphosphatemia. Although overall pyrophosphate production is higher during hyperphosphatemia, it was not sufficient to block calcium-phosphate deposition.

A growing body of evidence suggests that pyrophosphate is the predominant endogenous inhibitor of vascular calcification. The results of this study, along with previous findings, suggest that induction of pyrophosphate synthesis may be an easy and effective therapeutic strategy to inhibit vascular calcification associated with aging and other pathological conditions.
